# Fibroblastic Transformation of Corneal Keratocytes by Rac Inhibition is Modulated by Extracellular Matrix Structure and Stiffness

**DOI:** 10.3390/jfb6020222

**Published:** 2015-04-14

**Authors:** W. Matthew Petroll, Neema Lakshman

**Affiliations:** Department of Ophthalmology, UT Southwestern Medical Center, Dallas, TX 75390-9057, USA; E-Mail: neema.lakshman@utsouthwestern.edu

**Keywords:** extracellular matrix, biomechanics, corneal keratocytes, growth factors, Rho GTPases

## Abstract

The goal of this study was to investigate how alterations in extracellular matrix (ECM) biophysical properties modulate corneal keratocyte phenotypes in response to specific wound healing cytokines and Rho GTPases. Rabbit corneal keratocytes were plated within standard collagen matrices (2.5 mg/mL) or compressed collagen matrices (~100 mg/mL) and cultured in serum-free media, PDGF BB, IGF, FGF2 or TGFβ1, with or without the Rac1 inhibitor NSC23766 and/or the Rho kinase inhibitor Y-27632. After 1 to 4 days, cells were labeled for F-actin and imaged using confocal microscopy. Keratocytes within standard collagen matrices (which are highly compliant) maintained a dendritic phenotype following culture in serum-free media, PDGF, IGF and FGF, but developed stress fibers in TGFβ1. Keratocytes within compressed collagen (which has high stiffness and low porosity) maintained a dendritic phenotype following culture in serum-free media, PDGF and IGF, but developed stress fibers in both FGF and TGFβ1. The Rac inhibitor had no significant impact on growth factor responses in compliant matrices. Within compressed collagen matrices however, the Rac inhibitor induced fibroblastic transformation in serum-free media, PDGF and IGF. Fibroblast and myofibroblast transformation was blocked by Rho kinase inhibition. Overall, keratocyte growth factor responses appear to be regulated by both the interplay between Rho and Rac signaling, and the structural and mechanical properties of the ECM.

## 1. Introduction

The cornea is an optically clear tissue that forms the front surface of the eye, and accounts for approximately two-thirds of its refractive power. The corneal stroma makes up 90% of corneal thickness, and provides mechanical strength and structure to the tissue. The stroma consists of collagen sheets (lamellae) with regular packing and spacing of fibrils that is critical to maintenance of corneal transparency. Corneal stromal cells (keratocytes) reside between the collagen lamellae, and are responsible for secreting extracellular matrix (ECM) components required to maintain normal corneal structure and function [1,2,3]. From a mechanical standpoint, resting corneal keratocytes are considered quiescent; they do not express stress fibers or generate substantial contractile forces [4,5].

Keratocytes play an important role in mediating the corneal response to lacerating injury or refractive surgery [6]. During wound healing, quiescent corneal keratocytes adjacent to the injury transform into an activated, fibroblastic repair phenotype [7,8]. These activated fibroblasts proliferate, migrate into the provisional matrix, and generate the forces required for wound closure and/or ECM remodeling. In certain wound types, fibroblasts further differentiate into myofibroblasts, which generate even stronger forces and synthesize fibrotic ECM components [9,10]. These wound healing responses can cause a permanent reduction in corneal clarity by increasing light scattering, and can alter the refractive effect of vision correction surgeries such as photorefractive keratectomy (PRK) by changing corneal shape and/or thickness [11,12].

The Rho-family of small GTPases such as Rho, Rac, and Cdc42 mediate changes in cell mechanical activity in response to growth factors and other cytokines in a variety of cell types [13,14,15,16]. These GTP binding proteins function as molecular switches; alternating between the active GTP-bound state and the inactive GDP-bound state. In fibroblasts, activated Rho stimulates the formation of stress fibers and the development of focal contacts [17,18,19,20,21], and these cytoskeletal changes are dependent on actomyosin contraction [20,22]. Specifically, Rho is known to promote increased phosphorylation of myosin light chain via Rho-kinase (ROCK) inhibition of myosin light chain phosphatase (MLCPase), resulting in increased actin-myosin II based cell contractility [18,23]. In contrast to Rho, Rac induces cell spreading, via the creation of smaller focal complexes and actin polymerization [17,19,20,24,25].

In addition to growth factors and other biochemical stimuli, another factor that can modulate the keratocyte mechanical phenotype is the mechanical state of the ECM itself. For example, myofibroblast transformation of corneal keratocytes induced by TGFβ has been shown to be dependent on substrate or ECM stiffness [26,27], with stiffer substrates stimulating greater expression of α-smooth muscle actin. In addition, while FGF2 induces fibroblastic transformation of keratocytes plated on rigid 2-D substrates or within compressed 3-D collagen matrices, a quiescent mechanical phenotype is maintained in compliant (uncompressed) 3-D matrices [28]. In this study, we further investigate how alterations in extracellular matrix (ECM) biophysical properties can modulate corneal keratocyte phenotypes, by studying the effects of Rac inhibition on growth factor responses in 3-D matrices with different structural and mechanical properties.

## 2. Materials and Methods

### 2.1. Cells

Corneal keratocytes were isolated from rabbit eyes obtained from Pel Freez (Rogers, AR, USA) and cultured as previously described [5]. Cells were cultured in flasks with serum-free medium (basal medium) consisting of Dulbecco’s modified Eagle’s minimum essential medium with pyruvate (DMEM; Invtrogen, Carlsbad, CA, USA), supplemented with 1% RPMI vitamin mix (Sigma-Aldrich, St. Louis, MO, USA), 100 μM nonessential amino acids (Invitrogen, Carlsbad, CA, USA), 100 μg/mL ascorbic acid, and 1% penicillin/streptomycin/amphotericin B (Lonza Walkersville, Inc., Walkersville, MD, USA) to maintain the keratocyte phenotype [22]. For some experiments, previously published human corneal fibroblasts (HTK cells) were used [28,29]. HTK cells were maintained in tissue culture flasks with DMEM containing 10% FBS and supplemented with 1% penicillin/ streptomycin/ amphotericin B, then serum-starved in basal media for 7 days prior to an experiment.

### 2.2. Substrates

#### 2.2.1. Standard (Uncompressed) Collagen Matrices

Hydrated collagen matrices were prepared by mixing neutralized Type I Rat Tail collagen (BD Biosciences, San Jose, CA, USA) or bovine dermal collagen (Advanced BioMatrix, San Diego, CA, USA) with 10X MEM to achieve a final collagen concentration of 2.5 mg/mL [5]. A 50 μL of suspension of cells was then mixed with the above collagen solution. After adjusting the pH to 7.2 by addition of NaOH, 30 μL aliquots of the cell/collagen mixture (2 × 10^3^ or 5 × 10^4^ cells/matrix) were spread over a central 12 mm diameter circular area on Bioptechs culture dishes (Delta T; Bioptechs, Inc., Butler, PA, USA). The dishes were then placed in a humidified incubator for 30 min for polymerization. The matrices were then overlaid with 1.5 mL of serum-free media (basal media), and cultured as described below. All experiments were performed using duplicate samples, and repeated at least 2 times.

#### 2.2.2. Compressed Collagen Matrices

Compressed collagen matrices were prepared as described previously by Brown and coworkers [30,31,32]. Briefly, 10 mg/mL of Type I rat tail collagen was diluted to a final concentration of 2 mg/mL. After drop-wise neutralization with 1 M sodium hydroxide, a suspension of 2 × 10^4^ or 2 × 10^5^ keratocytes in 0.6 mL basal media was added to the collagen mixture. The solution containing cells and the collagen was poured into a 3 cm × 2 cm × 1 cm stainless steel mould and allowed to set for 30 min at 37 °C. In order to compact the matrices, a layer of nylon mesh (~50 μm mesh size) was placed on a double layer of filter paper. The matrices were placed on the nylon mesh, covered with a pane of glass and loaded with a 130 g stainless steel block for 5 min at room temperature. This process squeezes media out of the matrix and results in the formation of a flat, cell/collagen sheet with high mechanical stiffness. Following compression, 6 mm diameter buttons were punched out of the matrix using a trephine [32]. The matrices were then overlaid with 1.5 mL of serum-free media (basal media), and cultured as described below. Experiments were performed using duplicate samples, and repeated 3 times.

### 2.3. Culture Conditions

After 24 h to allow cell spreading, media was replaced with basal media supplemented with 50 ng/mL PDGF BB, 10 ng/mL IGF, 10 ng/mL FGF2, 10 ng/mL TGFβ1, or no growth factor (control) with or without the Rac1 inhibitor NSC23766 (50 μM) and/or the Rho kinase inhibitor Y-27632 (10 μM). Growth factor concentrations were determined from previous studies and represent the lowest concentration to give a maximal effect on changes in cell morphology and F-actin organization [32]. The inhibitor concentrations used have been shown to have maximal inhibition of the target while minimizing non-specific effects [33,34,35].

### 2.4. Imaging

After 1–4 days of culture in test media, cells were fixed using 3% paraformaldehyde in phosphate buffer for 15 min and permeabilized with 0.5% Triton X-100 in phosphate buffer for 3 min. To label F-actin, Alexa Fluor 488 phalloidin, or Alexa Fluor 546 phalloidin was used (1:20, Invitrogen). In some experiments, Rac1 activity was assessed by immunolabeling [36]. Following incubation in 1% BSA for 60 min to block non-specific binding, cells were incubated for 2 h in mouse monoclonal antibodies RacGTP (1:500, NewEast Biosciences, Malvern, PA, USA) in 1% BSA. Cells were then washed in buffer and incubated for 2 h in Alexa Fluor 488 conjugated goat anti-mouse secondary antibody. Constructs were imaged using laser scanning confocal microscopy (Leica SP2, Heidelberg, Germany) as previously described [34]. Stacks of optical sections (z-series) were acquired using a 63× water immersion objective (1.2 NA, 220 μm free working distance). Maximum intensity projections and color overlays were generated using MetaMorph.

## 3. Results

### 3.1. Cells within Uncompressed Collagen Matrices

**Figure 1:**
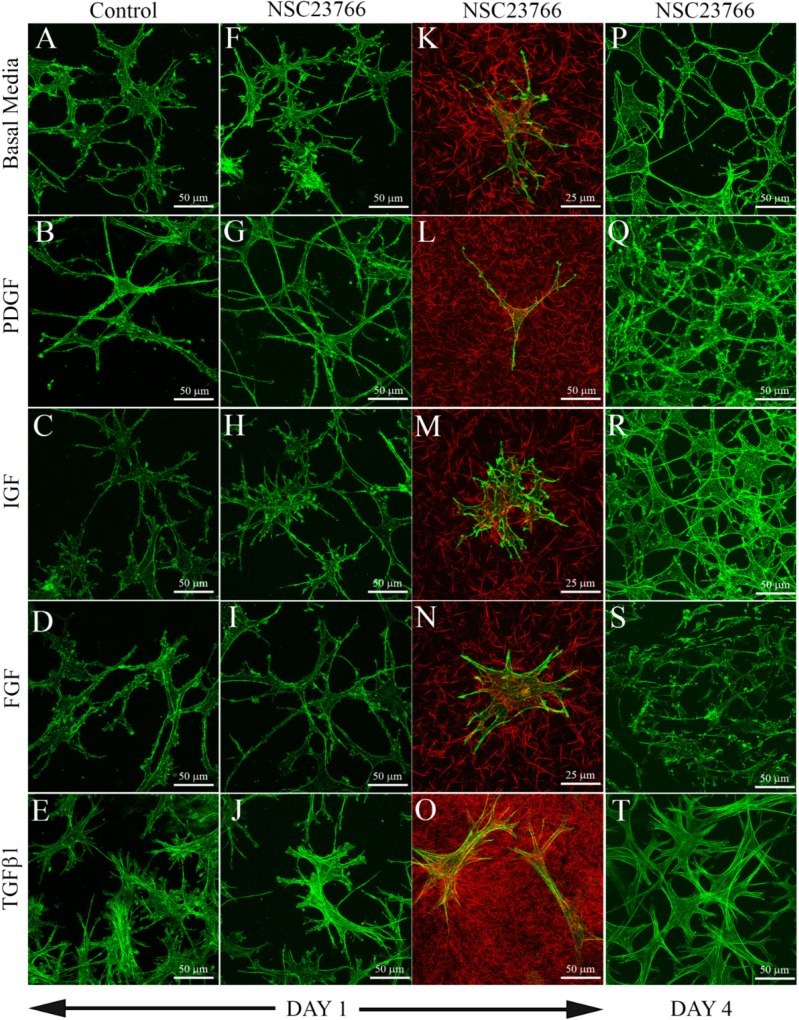
Maximum intensity projections of keratocytes plated within uncompressed hydrated collagen matrices, following 1 or 4 days of culture with the indicated growth factors, with or without the Rac1 inhibitor NSC23766 (50 μM). Green: F-actin, Red: Collagen (from confocal reflection imaging). Keratocytes within these collagen matrices (which are highly compliant) maintained a dendritic phenotype following culture in basal serum-free media (**A**), PDGF (**B**), IGF (**C**) and FGF (**D**), but developed stress fibers in TGFβ1 (**E**,**T**). Rac inhibition had no significant impact on growth factor responses in compliant matrices (**F**–**T**).

Consistent with previous studies, keratocytes cultured in serum free media (basal media) within uncompressed rat tail or bovine collagen matrices maintained a broad, convoluted cell body with numerous thin dendritic processes after both 1 day (Figure 1A) and 4 days (not shown) of culture. They had a cortical, membrane associated F-actin organization, with more concentrated labeling near the ends of cell processes. Stress fibers were rarely observed. Keratocytes exposed to PDGF BB (Figure 1B), IGF (Figure 1C) and FGF2 (Figure 1D) maintained this dendritic morphology, with cortical F-actin and no stress fibers. However, keratocytes treated with PDGF BB (Figure 1B) were much more elongated. In contrast, keratocytes treated with TGFβ1 (Figure 1E) lost dendritic processes and developed a more spread morphology. Within the cell body, F-actin filament bundles (stress fibers) were observed.

Addition of the Rac1 inhibitor NSC23766 had no visible impact on keratocyte growth factor responses within uncompressed 3-D collagen matrices at both 1 day (Figure 1F–O) and 4 days (Figure 1P–T) of culture. Cells in basal media, PDGF BB, IGF and FGF2 all maintained a dendritic morphology, whereas TGFβ induced stress fiber formation. Table 1 shows the percentage of fibroblastic cells under each condition tested, as indicated by the development of intracellular stress fibers and a loss of dendritic cell processes. It should be noted that within attached, uncompressed 3-D collagen matrices, the effective stiffness to which the cells are exposed increases near the bottom of the matrix, due to the rigid boundary condition (glass). Thus we only assessed cells that were between 15 and 100 μm above the glass. No significant differences in cell morphology were observed due to differences in the z-position of cells within this range. To further assess the mechanical phenotype of these cells, 3-D confocal images of isolated keratocytes from low cell density matrices were used to analyze the effects of growth factors and Rac inhibition on cell-induced matrix reorganization (Figure 1K–O). Fluorescent imaging was used to visualize F-actin and reflected light imaging was used to visualize collagen surrounding cells [34]. In general, minimal compaction and/or realignment of collagen fibrils was observed surrounding cells cultured in basal media, PDGF BB, FGF2, or IGF, both with (Figure 1K–N) and without (not shown) the addition of NSC23766. Note difference in scale bar for PDGF (Figure 1L), due to elongation of cells. In contrast, collagen surrounding cells in TGFβ1 was more compacted, both with (Figure 1O) and without (not shown) NSC23766.

**Table 1:** Percent of fibroblastic cells in uncompressed 3-D collagen extracellular matrix (ECM). *N* = 3 experiments; Mean ± Standard Deviation.

Culture media	1 day of culture		4 days of culture	
	Control	NSC23766	Control	NSC23766
Serum-Free	0 ± 0%	0 ± 0%	0 ± 0%	0 ± 0%
PDGF	0 ± 0%	0 ± 0%	0 ± 0%	3 ± 6%
IGF	0 ± 0%	5 ± 8%	0 ± 0%	5 ± 8%
FGF	0 ± 0%	0 ± 0%	0 ± 0%	0 ± 0%
TGFβ1	100 ± 0%	100 ± 0%	100 ± 0%	97 ± 6%

### 3.2. Cells on Bottom of Uncompressed Collagen Matrices

**Figure 2:**
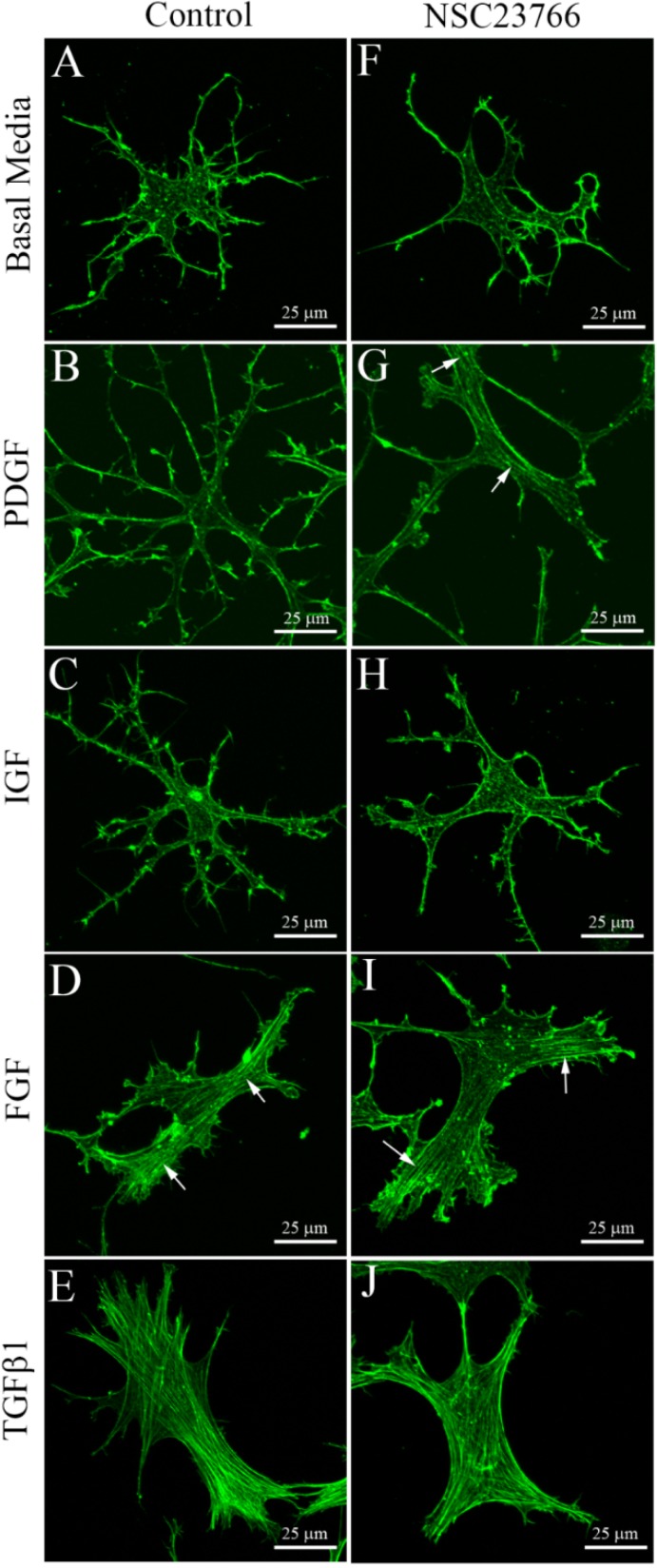
Maximum intensity projections of F-actin organization in keratocytes on the bottom of uncompressed hydrated bovine collagen matrices (attached to the rigid glass substrate) following 4 days of culture with the indicated growth factors, with or without the Rac1 inhibitor NSC23766 (50 μM). Keratocytes maintained a dendritic phenotype following culture in basal media (**A**), PDGF (**B**) and IGF (**C**), but developed stress fibers (arrows) in both FGF (**D**) and TGFβ1 (**E**). Rac inhibition induced fibroblastic transformation in PDGF (**G**), but had no effect in serum-free basal media (**F**) or IGF (**H**).

To assess the effect of substrate stiffness on the keratocyte response to growth factors and Rac inhibition, we studied cells on the bottom of the 3-D collagen matrices, where the effective stiffness is similar to that of a rigid (glass) substrate. This was accomplished by plating the unpolymerized bovine collagen solution immediately after adding the cell suspension, which allows the cells to sink to the bottom of bovine collagen and attach to the glass substrate (note that normally a 5 min pre-incubation is used to allow partial polymerization of the collagen, which prevents the cells from sinking to the bottom of the collagen solution). The growth factor responses observed on the bottom of 3-D collagen matrices were similar to our previously published results using keratocytes plated on 2-D collagen-coated glass substrates [27]. Specifically, in basal media, keratocytes developed a dendritic morphology with membrane associated F-actin labeling (Figure 2A). Keratocytes exposed to PDGF BB (Figure 2B) and IGF (Figure 2C) maintained this dendritic morphology, with cortical F-actin and no stress fibers. Keratocytes treated with PDGF BB (Figure 2B) were also more elongated. In contrast, FGF2 induced a switch from a dendritic morphology to a spread morphology, and prominent stress fiber bundles were observed in most cells (Figure 2D, arrows). TGFβ induced myofibroblast transformation, as indicated by loss of dendritic processes, development of stress fibers and α-smooth muscle actin labeling (not shown) (Figure 2E).

**Figure 3:**
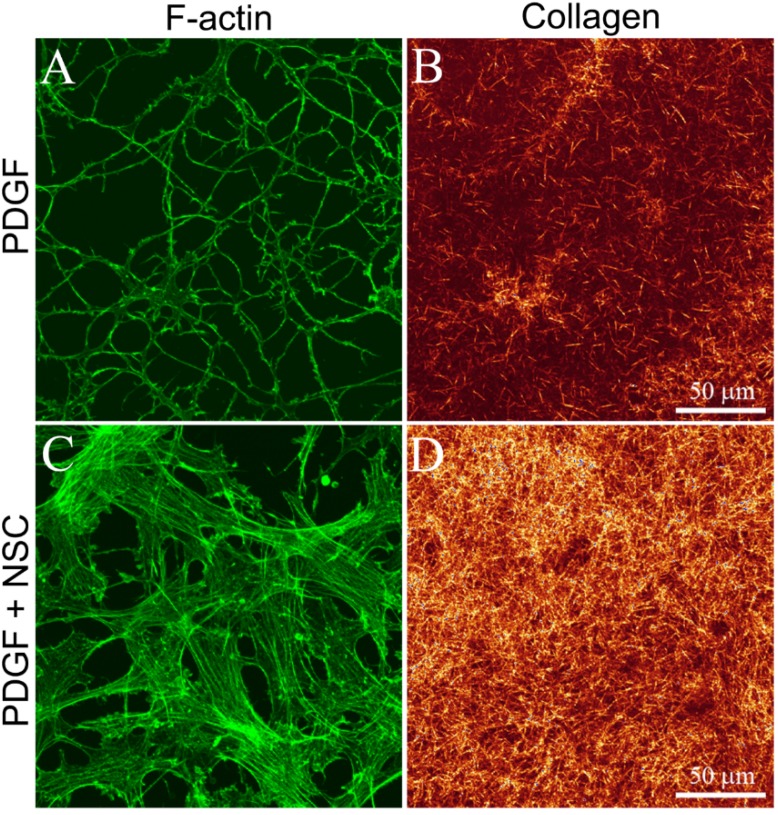
Maximum intensity projections of F-actin (**A**,**C**) and collagen (**B**,**D**) in cells on the bottom of hydrated bovine collagen matrices, following 4 days of culture in PDGF BB with or without the Rac1 inhibitor NSC23766 (50 μM). Cells develop stress fibers and compact the collagen on their apical surface following Rac1 inhibition.

Addition of the Rac1 inhibitor NSC23766 had no visible impact on the effects of basal media (Figure 2F), IGF (Figure 2H), FGF (Figure 2I) or TGFβ1 (Figure 2J) on cells at the bottom of 3-D collagen matrices. In contrast, for cells cultured in PDGF BB, inhibition of Rac induced a switch from a dendritic morphology to a spread morphology, and the development of stress fiber bundles in most cells (Figure 2G, arrows). To further assess this fibroblastic transformation, cell-induced matrix reorganization was assessed by imaging the collagen on the apical surface of the cells. Whereas minimal compaction of collagen fibrils was observed surrounding cells cultured in PDGF alone (Figure 3A,B), significant cell-induced compaction of the collagen was observed when NSC23766 was added to inhibit Rac activation (Figure 3C,D).

**Figure 4:**
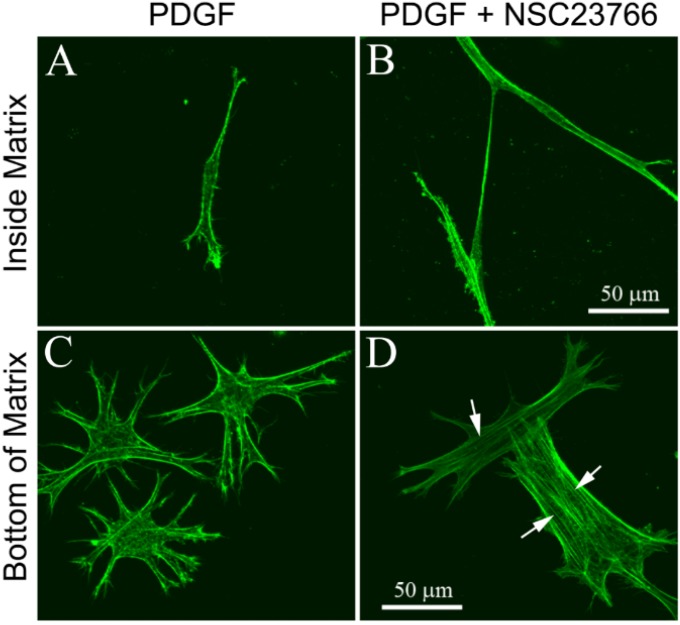
Maximum intensity projections of F-actin organization in serum-starved human corneal fibroblasts inside (**A**,**B**) or on the bottom (**C**,**D**) of uncompressed hydrated bovine collagen matrices, following 4 days of culture in PDGF BB with or without the Rac1 inhibitor NSC23766 (50 μM). Cells attached to the rigid glass substrate (bottom of matrix) lost their dendritic processes and developed stress fibers (arrows) when Rac1 was inhibited (D).

We also investigated the effect of substrate rigidity on serum-starved human corneal fibroblasts cultured in PDGF. As shown in Figure 4A, these cells normally have a bipolar morphology and do not develop stress fibers when plated within uncompressed collagen matrices. Corneal fibroblasts on the bottom of the 3-D matrix develop a more stellate morphology when cultured in PDGF, but still do not develop prominent stress fiber bundles (Figure 4C). Inhibition of Rac had no apparent impact on fibroblasts within the 3-D collagen matrix (Figure 4B); however, it induced a switch to a more spread morphology and the development of stress fibers in cells interacting with the glass substrate (Figure 4D, arrows).

In order to visualize changes in RacGTP in response to NSC23766, immunolabeling with an antibody specific to activated Rac was used [35,36]. As shown in Supplemental Figure S1, under serum-free conditions the dendritic processes showed the strongest labeling with the RacGTP antibody (Supplemental Figure S1A,B). Incubation with NSC23766 for 24 h resulted in a reduction in the intensity of RacGTP labeling of dendritic processes in some cells (Supplemental Figure S1C,D). Cells cultured in PDGF also showed strong RacGTP labeling of the dendritic cell processes, with much weaker labeling of the cell body (Supplemental Figure S1E,F). Incubation with NSC23766 and PDGF resulted in the development of stress fibers, and RacGTP labeling was observed in the cell body, but barely detectable in the cell processes (Supplemental Figure S1G,H). Control samples with no primary antibody showed very weak labeling of the cell body and no labeling of dendritic cell processes in both serum-free and PDGF culture conditions (not shown).

### 3.3. Cells within Compressed Collagen Matrices

**Figure 5:**
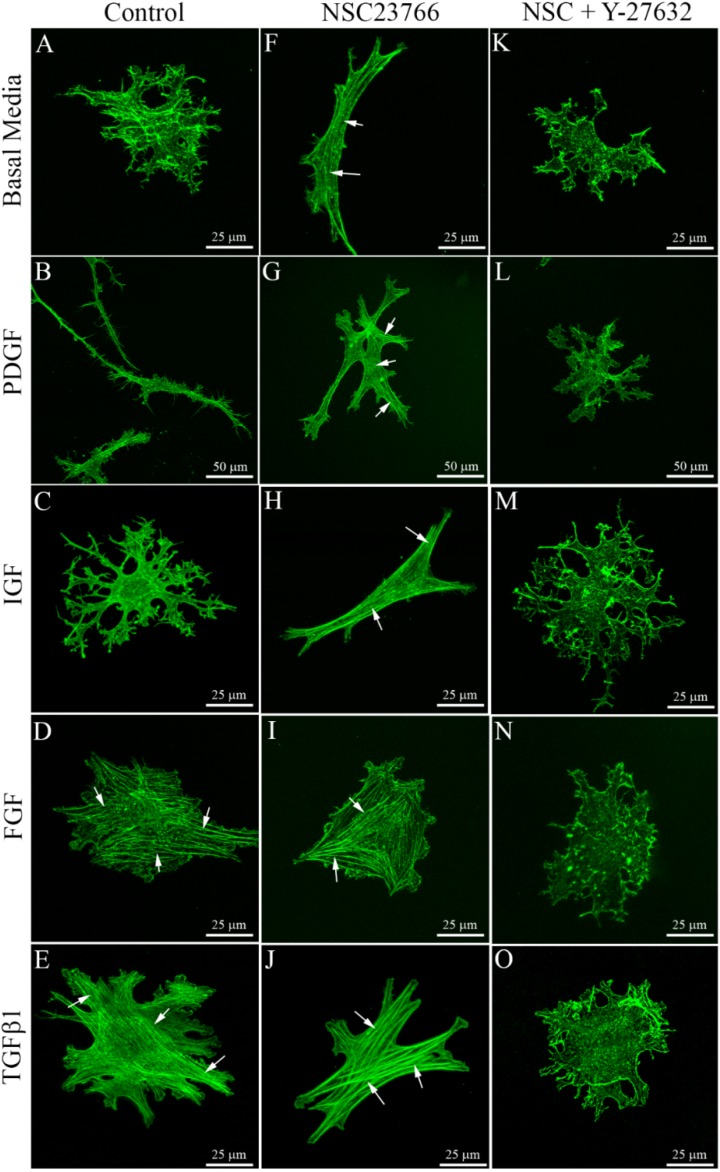
Maximum intensity projections of F-actin organization in keratocytes within compressed rat tail collagen matrices, following 4 days of culture with the indicated growth factors, with or without the Rac1 inhibitor NSC23766 (50 μM) and/or the Rho kinase inhibitor Y-27632 (10 μM). Keratocytes within compressed collagen (which has high stiffness and low porosity) maintained a dendritic phenotype following culture in basal serum-free media (**A**), PDGF (**B**) and IGF (**C**), but developed stress fibers (arrows) in both FGF (**D**) and TGFβ1 (**E**). Rac inhibition induced fibroblastic transformation in basal media (**F**), PDGF (**G**) and IGF (**H**). Fibroblast and myofibroblast transformation was blocked by Rho kinase inhibition (**K**–**O**).

In order to further investigate the role of ECM structure and stiffness on keratocyte responses, we plated cells within compressed collagen matrices, which provide a much stiffer 3-D culture environment than standard collagen matrices [30]. Keratocytes in compressed collagen matrices cultured in basal media (Figure 5A) or IGF (Figure 5C) generally had a stellate morphology with dendritic processes and did not develop stress fibers, as previously reported by us [27,32]. Culture in PDGF BB induced cell elongation (note different scale bars in Figure 5B, G and L), and stress fibers were only rarely observed (Figure 5B). In contrast, FGF2 induced a switch to a spread morphology, and prominent stress fiber bundles were consistently observed (Figure 5D, arrows) [27]. TGFβ also induced loss of dendritic processes and stress fiber formation (Figure 5E, arrows) [27]. Thus the growth factors induced a similar response in compressed collagen matrices as they did in cells on the bottom of uncompressed collagen ECM.

Addition of the Rac1 inhibitor NSC23766 had no visible impact on the effects of FGF2 (Figure 5I) and TGFβ1 (Figure 5J) on keratocytes within compressed collagen matrices. In contrast, for cells cultured in basal media (Figure 5F), PDGF BB (Figure 5G) or IGF (Figure 5H), inhibition of Rac induced a loss of dendritic cell processes and the development of stress fiber bundles in most cells (Figure 5, arrows), suggesting fibroblastic transformation. Table 2 shows the percentage of fibroblastic cells under each condition tested, as indicated by the development of intracellular stress fibers and a loss of dendritic cell processes.

We previously demonstrated that Rho kinase plays a central role in regulating corneal fibroblast contractility and matrix reorganization within standard 3-D matrices [34,37,38,39]. In 2-D culture, Rho kinase has also been shown to mediate fibroblastic and myofibroblastic transformation of keratocytes in response to FGF2 and TGFβ treatment, respectively [40]. In order to determine whether Rho kinase plays a role in transformation of keratocytes in compressed 3-D matrices, we used the established Rho kinase inhibitor Y-27632. Treatment with Y-27632 blocked the induction of stress fibers under all conditions evaluated (Figure 5K–O, Table 2).

**Table 2:** Percent of fibroblastic cells in compressed 3-D collagen ECM. *N* = 3 experiments; Mean ± Standard Deviation.

Culture media	1 day of culture			4 days of culture		
	Control	NSC23766	NSC + Y-27632	Control	NSC23766	NSC + Y-27632
Serum-Free	15 ± 13%	78 ± 25% *	0 ± 0% **	8 ± 10%	80 ± 19% *	0 ± 0% **
PDGF	7 ± 12%	58 ± 37% *	0 ± 0% **	9 ± 10%	85 ± 13% *	0 ± 0% **
IGF	19 ± 2%	57 ± 25% *	0 ± 0% **	12 ± 11%	81 ± 13% *	0 ± 0% **
FGF	95 ± 8%	100 ± 0%	0 ± 0% **	79 ± 26%	97 ± 6% *	0 ± 0% **
TGFβ1	100 ± 0%	100 ± 0%	0 ± 0% **	100 ± 0%	100 ± 0%	0 ± 0% **

* Significantly greater than Control (*P* < 0.05, two way ANOVA); ** Significantly less than NSC23766 (*P* < 0.05, two way ANOVA).

## 4. Discussion

Peptide growth factors present in the cornea and tear film, such as IGF, PDGF, FGF, IL-1α and TGFβ, are postulated to play an important role in modulating the keratocyte phenotype during corneal wound healing [41,42,43,44]. In cell culture, these growth factors differentially regulate keratocyte proliferation, cytoskeletal organization and ECM synthesis [1,45]. In addition to growth factors, mechanical signals from the ECM modulate phenotypes in a variety of cell types [46,47,48,49,50,51,52,53]. Increasing substrate stiffness can facilitate formation of actin stress fibers and focal adhesions in contractile cells [54,55,56], and both *in vitro* and *in vivo* studies have demonstrated that these structures tend to align along the tensile axis under anisotropic conditions [57,58,59,60,61,62,63,64]. Studies using corneal fibroblasts have shown similar differences in cell alignment, morphology, and matrix reorganization are observed between constrained (anisotropic) and unconstrained (isotropic) rectangular matrices [65].

The elastic modulus of newly polymerized 1–2 mg/mL hydrated collagen matrices measured by rheometry is generally less than 50 Pa [66,67,68], although the effective stiffness to which cells are exposed is likely higher in attached matrices due to the rigid boundary condition. By contrast, the stiffness of compressed collagen matrices has been reported to be on the order of 1 MPa [30,69], and the stiffness of glass used in tissue culture surfaces is >1 GPa [70]. We recently studied the effect of different ECM substrates on keratocyte mechanical phenotypes in response to growth factors present during wound healing [27]. Specifically, growth factor responses were compared using 2-D glass substrates, hydrated collagen matrices, and compressed collagen matrices. Consistent with the results of the current study (Table 3), it was demonstrated that keratocytes cultured in insulin growth factor (IGF) or PDGF BB maintain a quiescent mechanical phenotype over a range of ECM environments, including rigid 2-D glass substrates, hydrated collagen matrices and compressed collagen matrices [27]. Thus, the effects of these growth factors do not normally appear to be modulated by matrix density or stiffness. In contrast, FGF2 induced a contractile fibroblastic phenotype on more rigid substrates (2-D glass and compressed collagen matrices), while a quiescent mechanical phenotype was observed in compliant ECM. In hydrated 3-D collagen matrices, TGFβ1 & 2 only stimulated myofibroblast transformation at high cell density, where mechanical cross-talk between cells increases the tension within the matrix [22,27]. An increase in TGFβ-induced stress fiber formation and myofibroblast transformation of corneal keratocytes was observed within compressed 3-D collagen matrices; further demonstrating that increased substrate stiffness can enhance myofibroblast transformation of corneal keratocytes. Consistent with these results, Murphy and coworkers directly demonstrated that corneal fibroblasts grown compliant polyacrylamide substrates had fewer stress fibers and expressed significantly reduced amounts of α-SMA as compared cells plated on rigid 2-D substrates [26].

**Table 3:** Summary of conditions that induce fibroblastic transformation of corneal keratocytes.

Culture media	Uncompressed 3-D collagen ECM		Fibrillar collagen on glass (2-D)		Compressed 3-D collagen ECM	
	(↓ Stiffness, ↓ Collagen density)		(↑ Stiffness, ↓ Collagen density)		(↑ Stiffness, ↑ Collagen density)	
	Control	Rac1 inhibitor	Control	Rac1 inhibitor	Control	Rac1 inhibitor
Serum-Free	No	No	No	No	No	Yes
PDGF	No	No	No	Yes	No	Yes
IGF	No	No	No	No	No	Yes
FGF	No	No	Yes	Yes	Yes	Yes
TGFβ1	Yes	Yes	Yes	Yes	Yes	Yes

In the current study, the effects of Rac1 inhibition on the keratocyte phenotypes induced by PDGF BB, IGF, FGF2 and TGFβ1 was also assessed in different ECM environments. The Rho-family of GTPases, such as Rho and Rac, play a central role in the regulation of cell morphology, cytoskeletal organization and global contraction of 3-D collagen matrices. We previously demonstrated that Rho kinase plays a central role in mediating corneal fibroblast contractility and matrix reorganization within standard 3-D matrices [34,37,38,39], whereas PDGF-induced Rac activation stimulates spreading and migration of corneal keratocytes [29,71]. Thus we hypothesized that inhibition of Rac in corneal keratocytes would reduce cell spreading and increase cell contractility; *i.e.*, transform keratocytes from a mechanically quiescent, dendritic phenotype to a contractile, fibroblastic phenotype. To test this hypothesis, we used NSC23766, which inhibits Rac1 binding and activation via Rac-specific GEF Trio or Tiam 1, without altering RhoA or CDC42 binding or activation [33,72].

Interestingly, the effects of the Rac inhibitor were highly dependent on the structure and stiffness of the substrate used (Table 3). Rac1 inhibition had no impact on established growth factor induced responses within compliant ECM. This suggests that despite a partial reduction in Rac activation, the ratio of Rac/Rho activation remained high in these cells. However, when interacting with more rigid substrates (glass on bottom of uncompressed 3-D matrices or compressed collagen ECM), normally dendritic corneal keratocytes in PDGF BB developed a more compact morphology and developed stress fibers in response to Rac1 inhibition. A similar response was found in serum-starved human corneal fibroblasts. Consistent with this result, immunolabeling showed strong Rac GTP labeling localized to the dendritic cell processes following incubation in PDGF, but only cell body labeling following culture in NSC23766. Cells in serum-free media or IGF underwent fibroblastic transformation with Rac1 inhibition, but only in compressed ECM. The process of plastic compression reduces the height of the initial 2 mg/mL hydrated collagen matrix by a factor of approximately 50 (from 1 cm to ~200 μm), which results in a final collagen concentration of ~100 mg/mL. Thus, in addition to higher stiffness, these matrices have increased ligand density as compared to uncompressed collagen, and present more steric hindrance to cell spreading due to reduced porosity. ECM topography is also different between compressed and uncompressed matrices, and topographic signaling has also been shown to impact corneal fibroblast phenotypes [73]. Overall, these results demonstrate that changes in both structure and stiffness may influence signaling in response to Rac1 inhibition.

In 2-D culture, Rho kinase has also been shown to mediate fibroblast and myofibroblast transformation of keratocytes in response to FGF2 and TGFβ treatment, respectively [40]. In contrast, Rac stimulates cell spreading and migration [29,71,74], and has been shown to inhibit Rho activation in some cell types through p190RhoGAP [17,75,76]. In order to determine whether Rho kinase plays a role in fibroblastic transformation of keratocytes in compressed 3-D matrices following Rac1 inhibition, we used the established Rho kinase inhibitor Y-27632. Treatment with Y-27632 blocked the induction of stress fibers under all conditions evaluated. Thus it is possible that fibroblastic transformation induced by Rac1 inhibition may result from a shift in the balance between Rho and Rac activation. This could be a direct result of reduced Rac activation, and/or a loss of Rho inhibition by Rac1 [77]. Previous studies using human tenon fibroblasts in 3-D collagen matrices have demonstrated that Rac1 inhibition using NSC23766 or siRNA reduces cell protrusions and inhibits serum-induced global contraction of unrestrained (floating) collagen matrices [35]. This is consistent with other work demonstrating that fibroblast-induced contraction of unrestrained matrices is dependent on cell spreading, and Rho activation with LPA leads to retraction of cell processes without stress fiber formation [74]. In the current study we used attached collagen matrices, in which stress fibers can form under pro-contractile conditions, and global matrix contraction has been shown to be Rho kinase dependent.

Overall, these data suggest that keratocyte growth factor responses can regulated by both the interplay between Rho and Rac signaling, and the biophysical properties of the ECM. In the cornea, shifts in the distribution of mechanical tension can be induced by stromal injury or refractive surgery [12,78]. In addition, diseases such as keratoconus can lead to changes in stromal structure that result in reduced mechanical stiffness [79,80,81,82]. Thinning of the central cornea in keratoconus patients, results in a redistribution of tension within the stromal ECM [83]. In contrast, treatment of keratoconus with UV cross-linking increases corneal stromal stiffness [84]. Overall, cross talk between biochemical signaling and changes in ECM structure, stress and elasticity have the potential modulate both the acute and long-term responses of corneal keratocytes to a range of clinical conditions [85].

## Supplementary Files

Supplementary File 1
