# Supplementary material for: Fibroblastic Transformation of Corneal Keratocytes by Rac Inhibition is Modulated by Extracellular Matrix Structure and Stiffness
Source: J Funct Biomater. 2015 Apr 14;6(2):222–40. doi: 10.3390/jfb6020222 (PMC4493509; doi:10.3390/jfb6020222)
Supplement: Supplementary File 1 [file jfb-06-00222-s001.pdf]

J. Funct. Biomater. **2015**, *6*

OPEN ACCESS
Journal of
Functional
Biomaterials
ISSN 2079-4983
www.mdpi.com/journal/jfb

Supplementary Materials

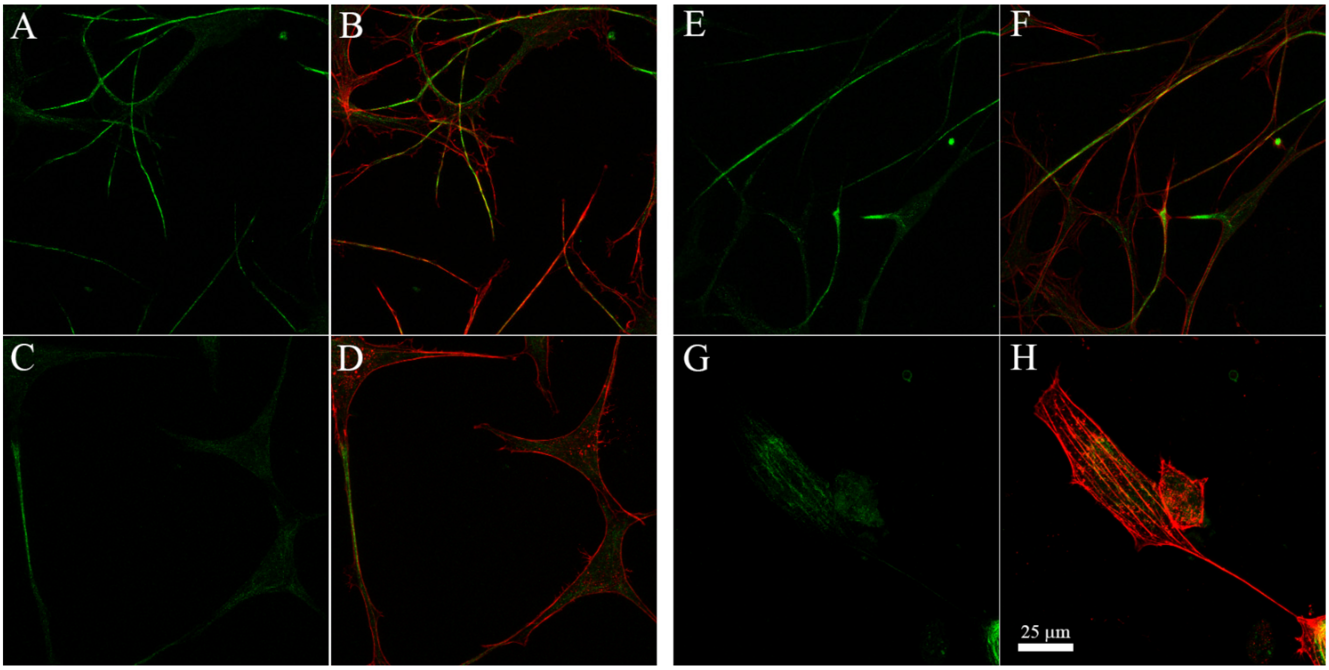

Figure S1. Rac GTP Immunolabeling of corneal keratcotyes on the bottom of 3-D collagen matrices. Green = Rac GTP, Red = F-actin. (**A**,**C**,**E**,**G**) show Rac GTP labeling. (**B**,**D**,**F**,**H**) show overlays of Rac GTP and F-actin labeling. (**A**,**B**) Cells cultured for 24 h in basal serum free media. Dendritic processes showed the strongest labeling with the RacGTP antibody; (**C**,**D**) Cells cultured in basal media plus NSC23766. Incubation with NSC23766 for 24 h resulted in a reduction in the intensity of RacGTP labeling of dendritic processes in some cells; (**E**,**F**) Cells cultured in PDGF for 24 h. Strong RacGTP labeling of the dendritic cell processes was observed, with much weaker labeling of the cell body; (**G**,**H**) Incubation with NSC23766 and PDGF resulted in the development of stress fibers, and RacGTP labeling was observed in the cell body, but barely detectable in the cell processes.
